# Influence of orbital symmetry on diffraction imaging with rescattering electron wave packets

**DOI:** 10.1038/ncomms11922

**Published:** 2016-06-22

**Authors:** M. G. Pullen, B. Wolter, A. -T. Le, M. Baudisch, M. Sclafani, H. Pires, C. D. Schröter, J. Ullrich, R. Moshammer, T. Pfeifer, C. D. Lin, J. Biegert

**Affiliations:** 1 ICFO—Institut de Ciencies Fotoniques, The Barcelona Institute of Science and Technology, 08860 Castelldefels (Barcelona), Spain; 2 J.R. Macdonald Laboratory, Physics Department, Kansas State University, Manhattan, Kansas 66506-2604, USA; 3 Max-Planck-Institut für Kernphysik, Saupfercheckweg 1, 69117 Heidelberg, Germany; 4 Physikalisch-Technische Bundesanstalt, Bundesallee 100, 38116 Braunschweig, Germany; 5 ICREA—Institució Catalana de Recerca i Estudis Avançats, 08010 Barcelona, Spain

## Abstract

The ability to directly follow and time-resolve the rearrangement of the nuclei within molecules is a frontier of science that requires atomic spatial and few-femtosecond temporal resolutions. While laser-induced electron diffraction can meet these requirements, it was recently concluded that molecules with particular orbital symmetries (such as *π*_g_) cannot be imaged using purely backscattering electron wave packets without molecular alignment. Here, we demonstrate, in direct contradiction to these findings, that the orientation and shape of molecular orbitals presents no impediment for retrieving molecular structure with adequate sampling of the momentum transfer space. We overcome previous issues by showcasing retrieval of the structure of randomly oriented O_2_ and C_2_H_2_ molecules, with *π*_g_ and *π*_u_ symmetries, respectively, and where their ionization probabilities do not maximize along their molecular axes. While this removes a serious bottleneck for laser-induced diffraction imaging, we find unexpectedly strong backscattering contributions from low-*Z* atoms.

## Abstract (web summary)

Laser-induced electron diffraction possesses sufficient resolution to resolve nuclear dynamics in molecules, but imaging was thought to depend on the orbital symmetries for the backscattering electrons. Here, the authors image the structure of randomly oriented O_2_ and C_2_H_2_ molecules and prove the contrary.

## Graphical abstract

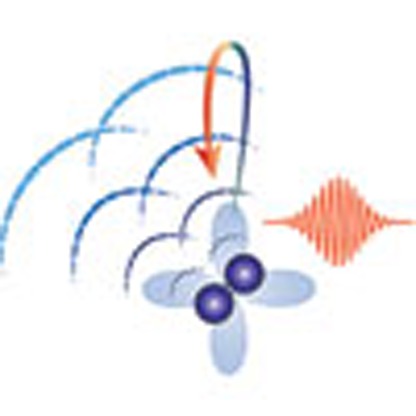

Knowledge about molecular structure is crucial to the understanding of complex chemical and biological systems. Gas-phase diffraction techniques^1^ provide well-established standards to image static structure. However, a deeper understanding of a system and its function only comes with temporally resolved measurements that enable one to observe the nature and timescales of how different components interact with each other. The advent of intense and ultrashort laser pulses prompted the development of techniques such as ultrafast electron diffraction^2^,^3^ and X-ray diffraction^4^, which are nowadays used to study molecular dynamics on previously inaccessible temporal scales, approaching a few hundred femtoseconds. However, to fully capture the entire dynamics of structural rearrangement, one needs to access the triggering events of a transformation, which demands access to the few-femtosecond temporal range. Not surprisingly, tremendous efforts are being made to access these timescales with current and emerging techniques^5^,^6^,^7^,^8^. Laser-induced electron diffraction (LIED)^9^ is a maturing technique that has already achieved few-femtosecond resolution on homonuclear diatomics,^10^ and it has recently been extended to polyatomic molecules^11^.

In LIED, the momentum distribution of strong-field-induced rescattered electrons is analysed as a function of the incident return momentum (*k*_r_) and scattering angle (*θ*_r_)^12^, and spatial resolutions of 5 pm are achievable when combining mid-infrared rescattering^13^ with kinematically complete detection^11^. While LIED makes use of the doubly differential scattering cross-section as a whole, a simpler analysis was recently presented by Xu *et al*.^14^, in which only backscattered (*θ*_r_≈180°) electron distributions are analysed. The appeal of using only a one-dimensional (1D) data set instead of the 3D doubly differential scattering cross-section lies in the faster data extraction procedure without the need for iterative algorithms. Moreover, such a Fourier analysis-based 1D version of LIED, FT-LIED, achieves much faster structural retrieval in a manner that is similar to ultrafast electron diffraction. However, when investigating the same simple diatomic species as in ref. 10, Xu *et al*. found that FT-LIED succeeded in retrieving the structure of N_2_, but failed for O_2_ due to a lack of structural information encoding in the respective backscattered electron distribution. They identified the failure of the FT-LIED methodology as being related to the ionization probability of the target molecule, which maximizes parallel with the bond axis for N_2_ but not for O_2_. From their findings, and based on their theoretical investigation, the authors concluded that in order to image molecular bonds with the FT-LIED technique the internuclear axis of interest must be aligned with the laser polarization direction. This conclusion has profound implications for LIED as it presents a strong impediment for further advancement of FT-LIED since most larger and complex molecules are not readily aligned (or oriented), and even those that can be would apparently require time-consuming tomographic techniques.

Here, we address the issue of the influence of the orbital symmetry of the target molecule and establish that it does not impede the retrieval of molecular structure. To demonstrate the utility of FT-LIED, we successfully image the structure of both O_2_ and C_2_H_2_ without molecular alignment and irrespective of the fact that molecular ionization is minimal along the molecular axis for both target species. Our results clearly show that the ionization angular dependence of the highest occupied molecular orbital (HOMO) does not prevent imaging of internuclear distances. This demonstrates that imaging is achievable from isotropic gas samples as routinely observed in ultrafast electron diffraction. We do, however, find that molecular structure can be obscured when the momentum transfer space is inadequately sampled. In addition, in our analysis we find unexpectedly strong scattering from hydrogen atoms. This observation presents a noticeable departure from the theoretical foundation of diffraction imaging, the independent atom model, which underestimates the contribution for the scattering conditions used here and warrants further theoretical investigations for the collision energies accessible to LIED.

## Results

### LIED imaging of randomly oriented molecules

**Figure 1:**
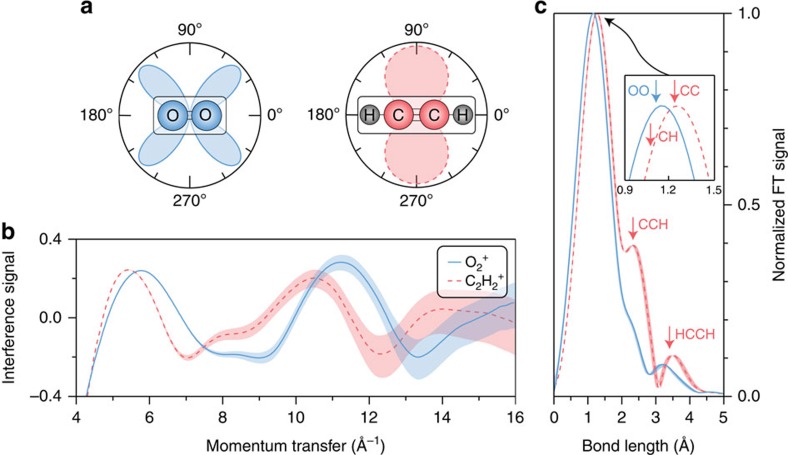
Extracting bond lengths independent of HOMO structure. (**a**) Simulated ionization probabilities of the O_2_ (blue solid curves throughout figure) and C_2_H_2_ (red dashed curves throughout figure) HOMOs as a function of the angle between the molecular axes and the laser polarization direction. (**b**) The interference signals obtained for O_2_ and C_2_H_2_ after ionization of the molecules by the 3.1-μm source. In this panel the shading denotes that the error bars are estimated via Poissonian statistics. (**c**) The result of Fourier-transforming the interference signals. The expected positions of the O_2_ and C_2_H_2_ cation internuclear distances are indicated. The inset shows a zoomed-in view around 1.2 Å, where a difference of about 0.14 Å can be observed between the main peaks of the two molecules. The shaded regions represent the estimated error in the extracted spectra resulting from the uncertainty in the value of the ponderomotive energy.

We first illustrate that the angle of maximum ionization from the HOMO does not need to coincide with the laser polarization axis to retrieve molecular structure. We choose as examples O_2_ (blue, solid throughout Fig. 1) and C_2_H_2_ (red, dashed throughout Fig. 1), whose HOMOs possess *π*_g_ and *π*_u_ symmetry, respectively, and in neither case do their ionization probabilities maximize parallel to the laser polarization direction (that is, at 0° or 180°), as shown in Fig. 1a. Electrons are predominantly emitted at 90° and 270° for C_2_H_2_ and at angles ∼45° away from parallel for O_2_. In order not to limit our investigation by the detection method, we resort to 3D electron-ion coincidence detection with a reaction microscope (see Methods for details) to measure the backscattered electron distributions. We have previously shown^5^ that our instrument's coincidence capabilities are important to provide high signal-to-noise data by discriminating backscattered electron distributions from different ionic species that are produced when ionizing the molecular targets. For a simple diatomic this issue is not necessarily a problem as only a few ionic components contribute. In the case of larger and more complex molecules, however, the number of detected ionic fragments will be large and their yields can be comparable to or greater than the main ion^15^.

Figure 1b shows the interference signals measured for O_2_^+^ and C_2_H_2_^+^ as a function of the momentum transfer experienced by the diffracted electrons (*q*=2*k*_r_sin(*θ*_r_/2)). We have isolated the molecular modulations from the experimental backscattered electron distributions by implementing empirical background subtraction (details are given in Supplementary Information in the section ‘Background subtraction'), in a manner analogous to ultrafast electron diffraction^1^. This method presents a number of important advantages compared with previous implementations of LIED. First, the structure of simple molecules can be determined without depending on theoretical calculations. Second, experimental data acquisition times will be reduced significantly as the backscattered electron distributions from partner atoms do not need to be measured. Third, and most importantly, all molecules are now potential targets as even those without partner atoms can be investigated. After background subtraction, it is observed that the resultant interference signals possess a number of extrema and zero-crossings, which are sensitive to molecular structure at the time of rescattering. The phase, frequency and amplitude are observed to be molecule dependent, which is a first indication that structural information is present. We note that, in general, the modulation amplitude of the experimental backscattered electron distributions is greater than that predicted by the independent atom model.

Fourier-transforming the measured interference signals results in a radial distribution spectrum that contains frequency components at the molecular internuclear distances. Before transformation a windowing function and zero padding are applied to the experimental data. The normalized spectra resulting from the Fourier transform of the interference signals in Fig. 1b are presented in Fig. 1c. Also indicated are the expected positions of the O_2_ and C_2_H_2_ cation internuclear distances^16^. The inset shows the region between 0.9–1.5 Å, where the anticipated O–O and C–C bond lengths of 1.12 and 1.25 Å, respectively, are located. The experimental peak positions are within 0.05 Å of these values. This finding clearly confirms that the bonds of molecules do not need to be aligned with the angle of maximum ionization rate in order to be successfully imaged.

We find that the main C_2_H_2_ cation peak near 1.3 Å is likely composed of two internuclear distances that are within <0.2 Å of each other and therefore not individually resolvable: the C–C bond mentioned above at 1.25 Å and the C–H (and H–C) bond at 1.08 Å (see inset of Fig. 1c). Due to the higher cross-section of C compared to H it is expected and observed that this peak should be shifted more towards the position of C–C than C–H. Interestingly and unexpectedly, a second and a third peak are also visible at distances of 2.33 and 3.51 Å and amplitudes of 0.40 and 0.11, respectively. The position of the second peak agrees with the cation C–C–H and H–C–C internuclear distance of 2.34 Å. This shows that scattering from the H atom is not negligible as expected from the independent atom model. We discard the third peak of the C_2_H_2_ spectrum due to low signal to noise even though it contains a signal corresponding to the expected H–C–C–H position. The exclusion of this region is supported by the fact that an artefact is found for O_2_ with similarly low amplitude at a similar position (3.21 Å).

### Limitations of the independent atom model

**Figure 2:**
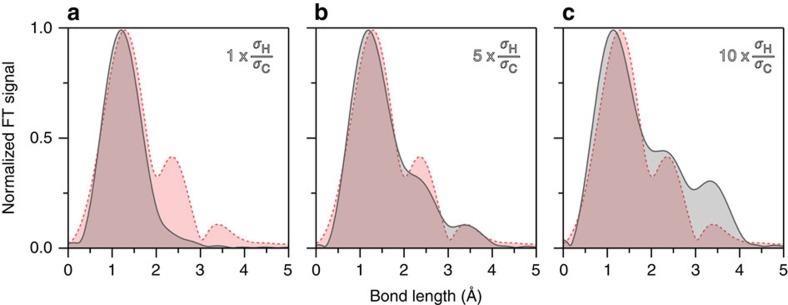
Limitations of the independent atom model. A comparison of the theoretical (grey, solid throughout figure) and experimental (red, dashed throughout figure) C_2_H_2_^+^ Fourier spectra. In (**a**) the independent atom model is unmodified while in (**b**) the *σ*_H_/*σ*_C_ ratio is increased by a factor of five and in (**c**) by a factor of ten. All spectra have been normalized to their maximum values.

The experimental diffraction data permitted to extract, for both species, O_2_ and C_2_H_2_, the most prominent contributions of heavy atoms corresponding to O–O and C–C distances, respectively. It is interesting, however, that we obtain clear signatures corresponding to the C–H and C–C–H bond distances. These experimental findings contradict calculations from the independent atom model, which predict that the differential cross-section for H atoms (*σ*_H_) should be one order of magnitude lower than for C atoms (*σ*_C_) for the typical scattering parameters used in LIED (*E*_r_=20–240 eV and *θ*_r_=180°). We compare our results to the independent atom model by calculating the expected differential cross-section as a function of returning electron energy at a constant scattering angle of *θ*_r_=180°. The result of the calculation for neutral C_2_H_2_ (grey, solid in all panels) is presented alongside our experimental results (red, dashed in all panels) in Fig. 2a. The independent atom model curve is dominated by one frequency component only, which can be observed at a position near 1.21 Å (neutral C–C bond length) after Fourier transformation. Thus, theory suggests that the influence of H atoms should not be observable in FT-LIED experiments for our experimentally accessible parameters. Note that the slight difference in the position of the main peak is due to the fact that neutral C_2_H_2_ is used in the simulation while the cation is measured in the experiment. If the independent atom model calculation is artificially modified by increasing the *σ*_H_/*σ*_C_ ratio by a factor of five, then other frequency components start to contribute to the interference signal. The most interesting finding is that the Fourier-transformed spectrum (Fig. 2b) now closely resembles the experimental one with a new contribution near 2.3 Å that is similar in magnitude to the measured one. Upon further increasing the *σ*_H_/*σ*_C_ ratio to a factor of ten, three Fourier peaks are visible and the similarity between the two curves clearly shows that the influence of H scattering can be observed in the experimental data (Fig. 2c). The amplitude of the 2.33 Å peak is about 40% of the main peak and therefore cannot be discarded. Its position is equal to the sum of the retrieved C–C plus C–H bond lengths, therefore corresponding to the distance of C–C–H. Moreover, the smaller Fourier amplitude of C–C–H compared with C–C is consistent with a larger separation, thus indicating that the C–C–H structure was successfully retrieved. We may use identical arguments to state that the small peak near 3.5 Å is the interference from the two H atoms. Based on the current independent atom model (Fig. 2a) it is hard to assess whether the experimental peak near 3.5 Å is related to molecular structure.

Our results suggest that the independent atom model does not fully describe electron scattering for the collision energies that are typical for FT-LIED. We note that differences between C_2_H_2_ independent atom model calculations and electron impact differential cross-section measurements have also been observed previously for similar scattering energies^17^. Using the entire doubly differential cross-section for the data analysis, the independent atom model (IAM) has shown excellent agreement with experimental C_2_H_2_ LIED data; however, this was at scattering angles ≤180° (ref. 11). While the apparent failure of the IAM is an important issue that should stimulate further investigations, both theoretical and experimental, our results show that the FT-LIED method continues to work irrespective of this failure. The larger relative contribution of hydrogen with respect to heavier atoms may become an advantage of LIED for studying dynamics involving proton migration, which plays a major role in many chemical reactions.

### Importance of sampling the momentum transfer space

We now address why the structure of O_2_ could not be imaged in ref. 14. In contrast with ultrafast electron diffraction, LIED makes use of the target molecule's own electrons for imaging. The LIED mechanism is based on strong-field tunnel ionization of the target molecule and rescattering off the target molecular ion. Field-free elastic scattering cross-sections are extracted by invoking the strong-field rescattering model, which can be fulfilled in the quasistatic, or deep tunnelling, regime. The unavoidable consequence of using a strong field to image molecules that possess relatively low ionization potentials (12.07 eV for O_2_ (ref. 18)) is the resulting number of strong-field processes such as fragmentation, Coulomb explosion and multiple ionization. Each of these processes has associated rescattering electrons carrying different momenta that obstruct the modulations observed in molecular backscattered electron distributions and therefore prohibit structural retrieval.

**Figure 3:**
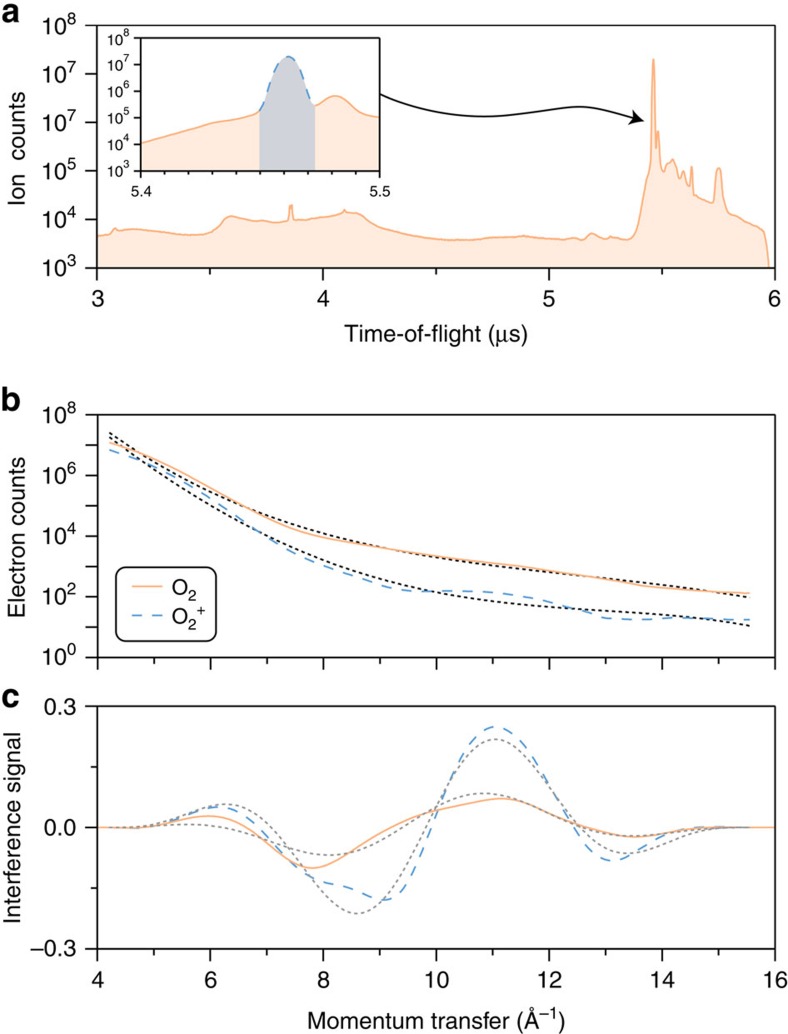
Importance of correctly sampling the momentum transfer space. (**a**) The ionic time-of-flight measured after the ionization of O_2_. The inset shows the small temporal range over which the O_2_^+^ ions are detected (blue shading). Other ions that are not of interest in this manuscript are also detected (orange shading). (**b**) The extracted backscattered electron distributions when electrons coincident with all ions (orange, solid) or only O_2_^+^ (blue, dashed) are utilized. Fourth-order polynomial fits are also presented (black, dotted). (**c**) The corresponding interference signals for O_2_^+^ (blue, dashed) and all (orange, solid) electrons. Single sinusoidal fits (grey, dotted) to the data show that the observable modulation has decreased by a factor of three.

To corroborate our explanation we illustrate the influence of these adverse processes on the image bearing backscattered electron distributions. Figure 3a shows ion time-of-flight data after ionization of O_2_ from which we had extracted backscattered electron distributions which were only associated to O_2_^+^ (Fig. 1b). Now we illustrate the importance of our selection of the momentum transfer space by eliminating coincidence conditions. A number of positively charged particles are detected, including the O_2_^+^ ion located near 5.5 μs (blue shading in inset). In Fig. 3b we compare the backscattered electron distributions extracted when using electrons coincident with O_2_^+^ only (blue, dashed—our conditions) and with all detected ions (orange, solid). Also shown are fourth order polynomial fits to the backscattered electron distributions (black, dotted). Two observations are evident: (1) an order of magnitude difference between the two backscattered electron distributions is visible over a large range of the spectrum, indicating that there are many contaminating electrons even for a simple species such as O_2_, and (2) the superimposed modulation is significantly enhanced when these contaminating electrons are excluded. The factor-of-three difference in modulation is more discernible after subtraction of the empirical background, as presented in Fig. 3c. In this case the signals are displayed with the windowing function already applied. Single sinusoidal fits to the data (grey, dotted) display how well the observed modulations can be represented by a single frequency and also help to quantify the decreased modulation.

The results in Fig. 3 show that even when using a relatively moderate laser intensity of 8.5 × 10^13^ W cm^−2^, a significant unwanted electron signal is present during mid-infrared strong-field ionization of molecular targets. In the case of O_2_ the desired electrons cannot be discriminated from this omnipresent unwanted signal without coincidence selection of the momentum transfer space. The peak laser intensity used to investigate O_2_ in ref. 14 was over 50% greater than the one used in this study. According to molecular Ammosov-Delone-Krainov simulations^19^ this would translate to an order of magnitude higher ionization rate. Thus, even more electrons would be generated from other strong-field processes, and it is these electrons that prohibited structural imaging of O_2_. In the case of a molecule such as N_2_, which was successfully imaged, its higher ionization potential of 15.58 eV (ref. 20) causes it to be harder to both ionize and fragment. Therefore the signal measured would most likely be dominated by N_2_^+^ electrons.

## Discussion

We have investigated whether molecular orientation has a prohibitive influence on the usage of backscattering electron wave packets to image molecular structure. Our investigation provides clear evidence that atomic-scale diffraction imaging does not require alignment of the target species with FT-LIED. Moreover, we use a simple empirically background-subtracted FT-LIED methodology with which we achieve imaging of two entirely different structures, the diatomic O_2_ and polyatomic C_2_H_2_ molecules. Overall, our results have important consequences for FT-LIED and atomic-scale imaging. First, the HOMO structure of an unaligned molecule poses no limitation on diffraction imaging with backscattering wave packets. Second, all molecules, rather than just those that can be aligned to a high degree, will be open to investigation. Lastly, we find that accurate bond lengths, particularly those involving atomic hydrogen, can still be accurately extracted using the FT-LIED method when the IAM begins to fail. This is an important advantage for FT-LIED that makes the investigation of time-resolved studies in complex molecules involving proton migration or isomerization possible. Further theoretical and experimental investigations with other hydrogen-rich molecules will be needed to firmly establish this important aspect of FT-LIED.

## Methods

### Light source

Our mid-infrared source is a home-built optical parametric chirped pulse amplification system that generates a number of wavelengths including a *λ*=3.1 μm output that has a duration of 75 fs full width at half maximum at a repetition rate of 160 kHz (ref. 21). The high repetition rate more than compensates for the unfavourable *λ*^−4^ scaling of strong-field induced rescattering^22^. A 50-mm-focal-length mirror focuses the 3.1-μm radiation to a spot size of about 6–7 μm, which results in a peak intensity of about *I*=8.5 × 10^13^ W cm^−2^. The intensity was estimated using a variety of techniques. Such a peak intensity results in a Keldysh parameter of *γ*≈0.3 and a ponderomotive energy of *U*_P_=75 eV, which corresponds to maximum classical return and backscattered electron energies of *E*_ret,max_=3.17*U*_P_≈240 eV and *E*_back,max_=10*U*_P_≈750 eV, respectively. Such high-energy photoelectrons are those that scatter near the nucleus of each atom, where the potential from the core dominates the influence from the outer-shell electron distribution^23^.

### Detection system

The laser focus intersects with a rotationally cold gas jet (*T*≤100 K for both molecules) that passes through two skimmer stages. Both gases are of high purity. The interaction takes place in an ultra-high vacuum chamber that has a base pressure 10^−11^ mbar without gas load and that houses a reaction microscope detection system^24^. The reaction microscope is able to detect all charged products of the interaction in full coincidence and with 3D momentum information. The magnetic and electric field strengths (12.9 G and 550 V, respectively) were set such that no ‘dark regions' (otherwise known as nodes) were located in the half of the electron momentum distribution used during data analysis. There are two reasons why the coincidence capabilities of the reaction microscope are of importance to this study: (1) the capacity to isolate the electrons of interest (i.e., those correlated to O_2_^+^ or C_2_H_2_^+^ only) from those corresponding to other strong-field processes so that uncontaminated backscattered electron distributions can be obtained; and (2) the ability to emulate a time-of-flight spectrometer by extracting backscattered electron distributions that include electronic contributions related to all ion fragments created during the experiment.

### Data availability

The data that support the findings of this study are available from the corresponding author upon request.

## Additional information

**How to cite this article:** Pullen, M. G. *et al*. Influence of orbital symmetry on diffraction imaging with rescattering electron wave packets. *Nat. Commun.* 7:11922 doi: 10.1038/ncomms11922 (2016).

## Supplementary Material

Supplementary Information Supplementary Figures 1-3 and Supplementary Discussion.
