# Supplementary material for: Influence of orbital symmetry on diffraction imaging with rescattering electron wave packets
Source: Nat Commun. 2016 Jun 22;7:11922. doi: 10.1038/ncomms11922 (PMC4917885; doi:10.1038/ncomms11922)
Supplement: Supplementary Information — Supplementary Figures 1-3 and Supplementary Discussion. [file ncomms11922-s1.pdf]

Supplementary Figures

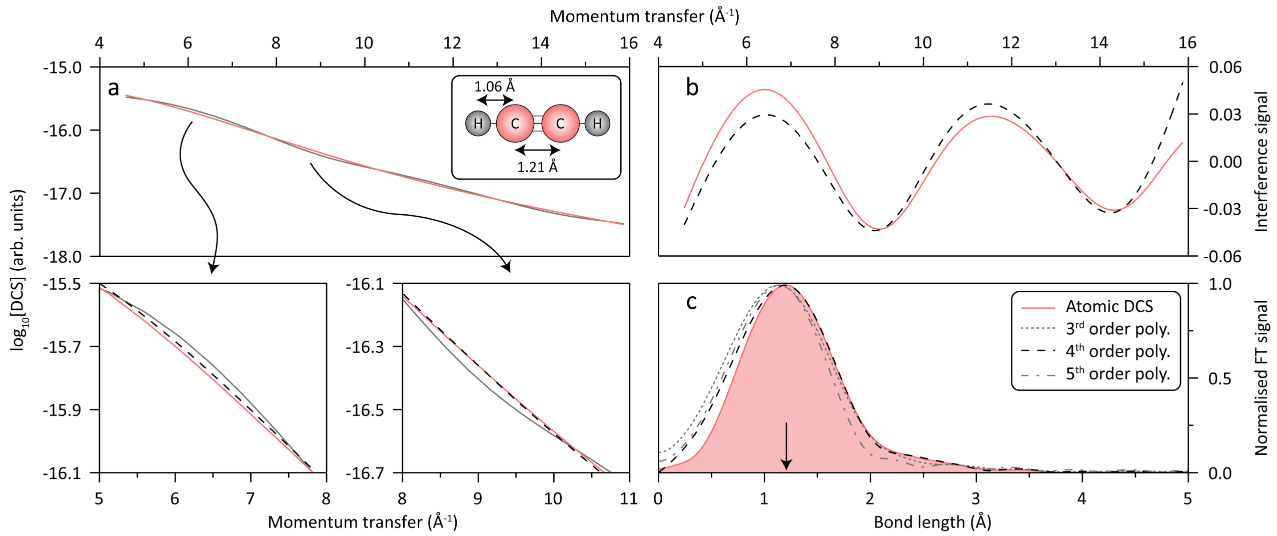

Supplementary Figure 1: Isolation of the interference signal. (a) The calculated molecular (grey solid) and atomic (red solid curves throughout figure) DCSs. The bottom panels show zoomed in regions where modulations of the atomic DCS about the molecular DCS are observed. A fourth order polynomial fit of the molecular DCS (black dashed curves throughout figure) is also shown in each panel. (b) The calculated interference signals (interference signals) after subtraction of the atomic DCS and the polynomial fit. (c) The resultant Fourier transforms of the interference signals alongside the results from third (dotted grey) and fifth (dot-dashed grey) order polynomial fits.

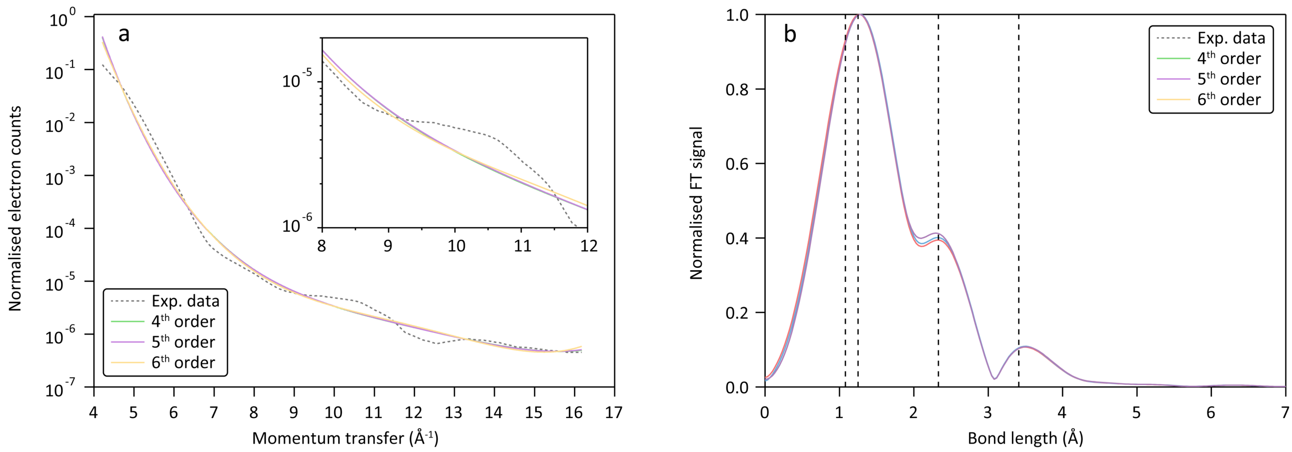

Supplementary Figure 2: Polynomial background subtraction. (a) The detected backscattered electron distribution corresponding to the $C_2H_2^+$ ion. Polynomial fits of 4th, 5th and 6th order are also presented. The inset shows a zoomed in view between 8 - 12 Å^{-1} that highlights how the fits lie almost on top of each other. (b) The result of Fourier transforming the interference signals obtained after subtracting the polynomial fits in (a). Very similar behavior is found when investigating O_2.

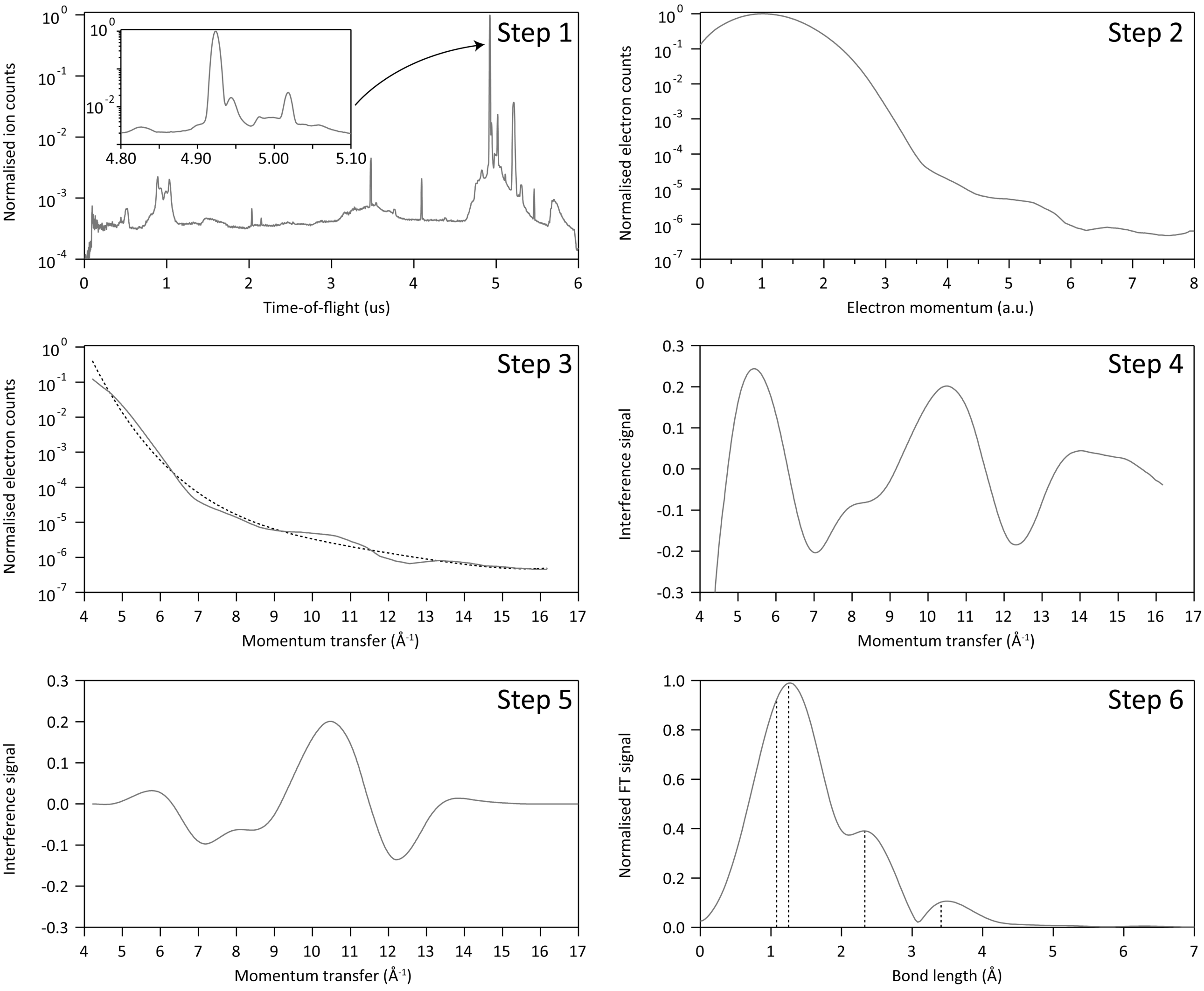

Supplementary Figure 3: Data reduction procedure. The steps taken to arrive at the $C_2H_2^+$ bond length spectrum from the experimental data. The same steps are taken to analyse the O2 data. See the text for more details.

Supplementary Discussion

Isolation of molecular information from backscattering electron distributions.

A crucial step in extracting molecular structure imprinted in experimentally measured molecular backscattered electron distributions (ρ_E) the superimposed modulations need to be isolated from the overall distribution by subtracting the monotonically decreasing 'background'. We therefore outline our procedure before applying it to experimental results. The resulting curve can be called the interference signal and an analogous parameter is used in conventional electron diffraction [1]. In principle, the interference signal can be calculated by subtracting either theoretical simulations or the backscattered electron distributions from atoms with similar ionisation potentials that were measured under the same experimental conditions. Here, and for the first time, we calculate the interference signal from LIED by subtracting an empirically determined background (ρ_B) from the logarithm of ρ_E,

$$\rho = \log_{10}(\rho_E) - \log_{10}(\rho_B) = \log_{10}(\rho_E/\rho_B).$$

We test our empirical background fitting procedure on independent atom model based simulations that were calculated following procedures that have been outlined previously [11, 23]. Since the independent atom model does not need to take into account the laser-field, we emulate experimental backscattered electron distributions by simply calculating differential cross-sections (DCS) as a function of returning electron energy at a constant scattering angle of ϑ_r = 180°. The calculated molecular DCS (grey, solid) of C_2H_2 is observed to modulate about the corresponding atomic DCS (red, solid) in Supplementary Fig. 1a. These modulations are due to the coherent interference of scattered waves from each atom in the molecule. The bottom two panels show zoomed in regions where the modulations are more visible. The atomic term can be considered equivalent to the background ρ_B mentioned above. Also shown is a fourth order polynomial fit (black dashed) to the molecular DCS that very closely follows the atomic term. The interference signals resulting from the subtraction of the atomic DCS (red, solid) and the empirical fit (black, dashed) are presented in Supplementary Fig. 1b. The main features of the atomic subtracted interference signal are reproduced by the empirical fit. The normalised Fourier transforms of these signals are presented in Supplementary Fig. 1c alongside the results for when third (grey, dotted) and fifth (grey, dot-dashed) order polynomials are utilised. The most important observation is that the peak of the fundamental frequency component is within 0.05 Å for all curves. This shows that empirical background subtraction based on polynomial fitting is a robust and accurate method to isolate molecular modulations in FT-LIED. We also tested this polynomial background subtraction procedure on the data presented in Fig. 1a & Supplementary Fig. 3c of Ref. [14] and our results were consistent with those published.

Background subtraction

The robustness of the background subtraction methodology is presented in Supplementary Fig. 2. Thorough tests were performed on the experimental data to ensure that the results of the background fitting algorithm were consistent. In Supplementary Fig. 2a the experimentally measured backscattered electron distribution from C_2H_2 is presented alongside lines of best fit for 4th, 5th and 6th order polynomials. A zoomed in version between 8-12 $Å^{-1}$ shows that these fits lie mostly on top of each other. It was found that these polynomial orders gave almost identical results for both O_2 and C_2H_2, which highlights the reliability of the background subtraction procedure. The resultant Fourier transformed spectra are presented in Supplementary Fig. 2b where almost identical results are observed. We find that polynomial fits below 3rd order do not adequately remove the 'background' signal and fits above 6th order start to remove the real signal.

Experimental data analysis

The procedure to get from the raw experimental data to the bond length spectrum is outlined in Supplementary Fig. 3. We focus on the C_2H_2 case here but the method is the same for O_2.

Step 1: The ionic time-of-flight shows a variety of positively charged particles detected after single and multiple ionisation as well as fragmentation. The inset shows a zoomed in view of the C_2H_2 cation peak.

Step 2: The electrons associated with the C_2H_2 cation peak are extracted as a function of their final detected momentum. Only electrons that scatter within a ±4° cone around the laser polarisation are used in the data analysis.

Step 3: The return momenta of the electrons are calculated from the final momenta using classical trajectory analysis combined with an estimate of the ponderomotive energy of U_P = 75 eV. Only the portion of the spectra where modulations are found is used. A polynomial is fitted (dotted curve) to the experimental data (see Supplementary Fig. 3 for more details).
Step 4: The polynomial line of best fit is subtracted from the backscattered electron distribution to isolate the molecular interference signal.
Step 5: A windowing function is applied to the interference signal to reduce spectral leakage and then zero padding is introduced to interpolate in the Fourier domain.
Step 6: After Fourier transformation of the interference signal the bond length spectrum is obtained. Peaks near the expected bond lengths are clearly observed.
